# The prognostic value of co-expression of stemness markers CD44 and CD133 in endometrial cancer

**DOI:** 10.3389/fonc.2024.1338908

**Published:** 2024-04-19

**Authors:** Peng Jiang, Chenfan Tian, Yunfeng Zheng, Chunxia Gong, Jinyu Wang, Ying Liu

**Affiliations:** ^1^ Department of Gynecology, The First Affiliated Hospital of Chongqing Medical University, Chongqing, China; ^2^ Department of Gynecology, Women and Children’s Hospital of Chongqing Medical University, Chongqing, China

**Keywords:** endometrial cancer, CD44, CD133, co-expression, prognostic value

## Abstract

**Objective:**

The purpose of this study was to investigate the correlation between stemness markers (CD44 and CD133) and clinical pathological features, and to further explore the prognostic value of co-expression of CD44 & CD133 in endometrial cancer (EC).

**Methods:**

Clinical data of stage I-III EC patients who underwent initial surgical treatment at two large tertiary medical centers from 2015 to 2020 were retrospectively collected. Cohen’s kappa coefficient was used to show the consistency of the expression between CD44 and CD133. The correlation between co-expression of CD44 & CD133 and prognosis of EC patients was explored using univariate and multivariate Cox regression analysis. Then, the prognosis models for early-stage (stage I-II) EC patients were constructed. Finally, stratified analysis was performed for EC patients in high-intermediate-risk and high-risk groups, Kaplan-Meier analysis was used to compare the survival differences between patients with and without adjuvant therapy in different co-expression states (low expression, mixed expression, high expression) of CD44 & CD133.

**Results:**

A total of 1168 EC patients were included in this study. The consistency of the expression between CD44 and CD133 was 70.5%, the kappa coefficient was 0.384. High expression of CD44 & CD133 was associated with early FIGO stage (P=0.017), superficial myometrial invasion (P=0.017), and negative lymphatic vessel space invasion (P=0.017). Cox regression analysis showed that the co-expression of CD44 & CD133 was significantly correlated with the prognosis of early-stage (stage I-II) patients (P=0.001 for recurrence and P=0.005 for death). Based on this, the nomogram models were successfully constructed to predict the prognosis of early-stage EC patients. Meanwhile, Kaplan-Meier analysis showed that patients with adjuvant therapy had a better overall prognosis than those without adjuvant therapy in high-intermediate-risk and high-risk groups. However, there was no statistically significant difference in survival between patients with and without adjuvant therapy in high expression of CD44 & CD133 group (P=0.681 for recurrence, P=0.621 for death).

**Conclusion:**

High expression of CD44 & CD133 was closely related to the adverse prognosis of early-stage EC patients. Meanwhile, patients with high expression of CD44 & CD133 may not be able to achieve significant survival benefits from adjuvant therapy.

## 1 Introduction

Endometrial cancer (EC) is one of the three major malignant tumors in the female reproductive system (1). The overall prognosis of the patients is favorable; however, a subset of individuals experiences adverse outcomes due to tumor recurrence, metastasis, and the development of drug resistance, which are associated with an unfavorable prognosis. Empirical evidence indicates a strong correlation between these negative prognostic factors and the stemness of the tumor cells (2). CD44 and CD133 are recognized surface markers of tumor stem cells (3). Among them, CD44 is a non-kinase antigen that is expressed on various cell types of embryonic stem cells (4). Recently, CD44 has been used to identify different cancer stem cells (CSCs), such as lung cancer, breast cancer, colon cancer, blood system cancer and others (5). CD133 is a five transmembrane glycoprotein first discovered in 1997 and expressed on hematopoietic stem cells and progenitor cells produced in blood, fetal liver, and bone marrow (3). Numerous investigations have demonstrated that CD133 serves as a promising marker for cancer stem cells (CSCs) and is present across a spectrum of tumor types. This includes, but is not limited to, malignancies of the breast, brain, kidney, lung, pancreas, and ovaries (5).

It has been reported in the literature that tumor cells with CD44-high & CD133-high exhibit stronger tumor stem properties than those with CD44-low & CD133-high or CD44-high & CD133-low, such as higher tumorigenicity, stronger self-renewal ability, stronger drug resistance, and more stem related genes (6). Therefore, the co-expression of CD44 & CD133 has been extensively utilized for the isolation and identification of cancer stem cells in a variety of malignant tumors, including but not limited to colorectal cancer, non-small cell lung cancer, ovarian cancer, prostate cancer, and gallbladder cancer (7). In EC, CD44 and CD133 are also used as surface markers of tumor stem cells in most studies (3, 8, 9). In a recently published study, we also successfully induced EC stem-like cells with high expression of CD44 & CD133 (10). However, most of these studies are still in the laboratory stage, and the exploration on the value of CD44 and CD133 in clinical application of EC are still very rare. Consequently, the objective of this study is to elucidate the association between the co-expression of CD44 & CD133 with clinicopathological characteristics, and to assess the prognostic implications of co-expression of CD44 & CD133 in EC based on a dual center study cohort.

## 2 Materials and methods

### 2.1 Patient cohort, treatment, and follow-up

Retrospective collection of clinical and pathological data of stage I-III [according to 2009 FIGO guidelines (11)] EC patients who underwent initial surgical treatment at two large tertiary medical centers (the First Affiliated Hospital of Chongqing Medical University & the Women and Children’s Hospital of Chongqing Medical University) from January 2015 to June 2020 was performed. The inclusion criteria for patients were as follows: (1) The patient underwent standard comprehensive staging surgery, including at least abdominal total hysterectomy + bilateral salpingo-oophorectomy + lymph node assessment (lymph node assessment mainly includes pelvic lymph node resection ± paraaortic lymph node resection) (12); (2) The final pathological diagnosis of the patient after surgery was endometrial cancer, and the pathological stage was FIGO stage I-III (Most stage IV patients did not receive surgical treatment and were unable to obtain postoperative pathological specimens. Meanwhile, stage IV patients had distant metastasis before treatment, which affected the subsequent assessment of patient recurrence. Therefore, stage IV patients were not included in this study). The exclusion criteria were as follows: (1) Patients with incomplete medical records; (2) Receiving adjuvant therapy before surgery; (3) Patients with other malignancies; (4) Loss of follow-up.

All patients were recommended to receive corresponding adjuvant treatment after surgery according to corresponding guidelines and multidisciplinary discussions (specific adjuvant treatment plans can be found in previous published articles (13, 14)). The implementation of the final adjuvant treatment plan was driven by the professional advice of clinical doctors and the personal wishes of patients, including follow-up, radiotherapy alone, chemotherapy alone, and the combination of radiotherapy and chemotherapy (chemoradiotherapy).

The follow-up plan of patients was carried out according to the corresponding guidelines: in short, follow-up was performed every 3 months for the first 2 years after surgery, every 6 months for the next 3 years, and annually thereafter (15). The follow-up plans included regular physical examination and necessary auxiliary examinations, including serological examination (screening of tumor markers), imaging examination (B-ultrasound, CT, PET-CT, and MRI), and pathological biopsy (13). During the follow-up period, two or more gynecological oncologists would confirm EC recurrence (including vaginal stump recurrence, central pelvic region recurrence, upper para-aortic lymph node metastases, peritoneal metastases, and metastases to other organs) based on the above examination results and strive to obtain pathological support. Recurrence-free survival (RFS) was defined as the time from the surgical date to the confirmed recurrence date, and overall survival (OS) was defined as the time from the surgical date to the death (16). The follow-up deadline for this study was August 2023, and each patient has been guaranteed a follow-up period of more than 3 years.

### 2.2 Postoperative pathological examination and immunohistochemical analysis

The postoperative surgical specimens of the patient were immediately fixed with standard 10% neutral formalin tissue fixative after removal from the body and sent to the Pathological Experimental Center of Chongqing Medical University for subsequent processing within 24 to 48 hours, including dehydration, paraffin embedding, sectioning, H&E staining, and immunohistochemical analysis. The pathological results (histological type and grade, myometrial invasion, cervical stromal invasion, lymphatic vessel space invasion and lymph node metastasis, etc.) was evaluated by professional pathologists. Grade 1 (G1) and grade 2 (G2) endometrial adenocarcinoma was defined as the pathological type I of EC, while grade 3 (G3) endometrial adenocarcinoma and non-endometrial adenocarcinoma (including serous carcinoma, clear cell carcinoma, and other special histological types) was defined as pathological type II (17).

Immunohistochemical analysis of P53, CD44 and CD133 were performed by using a fully automatic immunohistochemistry staining instrument (Leica Bond Max, Milton Keynes, UK) according to a uniform and optimized immunohistochemical protocol. The following antibodies were used as primary antibodies for the immunohistochemical analysis: P53 (MAB-0674, Maixin Biotech, China), CD44 (15675-1-AP, Proteintech, China) and CD133 (18470-1-AP, Proteintech, China) (specific immunohistochemical analysis step can be found in previous published articles (14, 16)). The central regions of 5 tumor sections were randomly selected and the immunohistochemical results of P53, CD44 and CD133 were evaluated based on the staining intensity (weak staining, medium staining, and strong staining) and the proportion of positive tumor cells under a 40 (objective) ×10 (eyepiece) magnification field.

**Figure 1:**
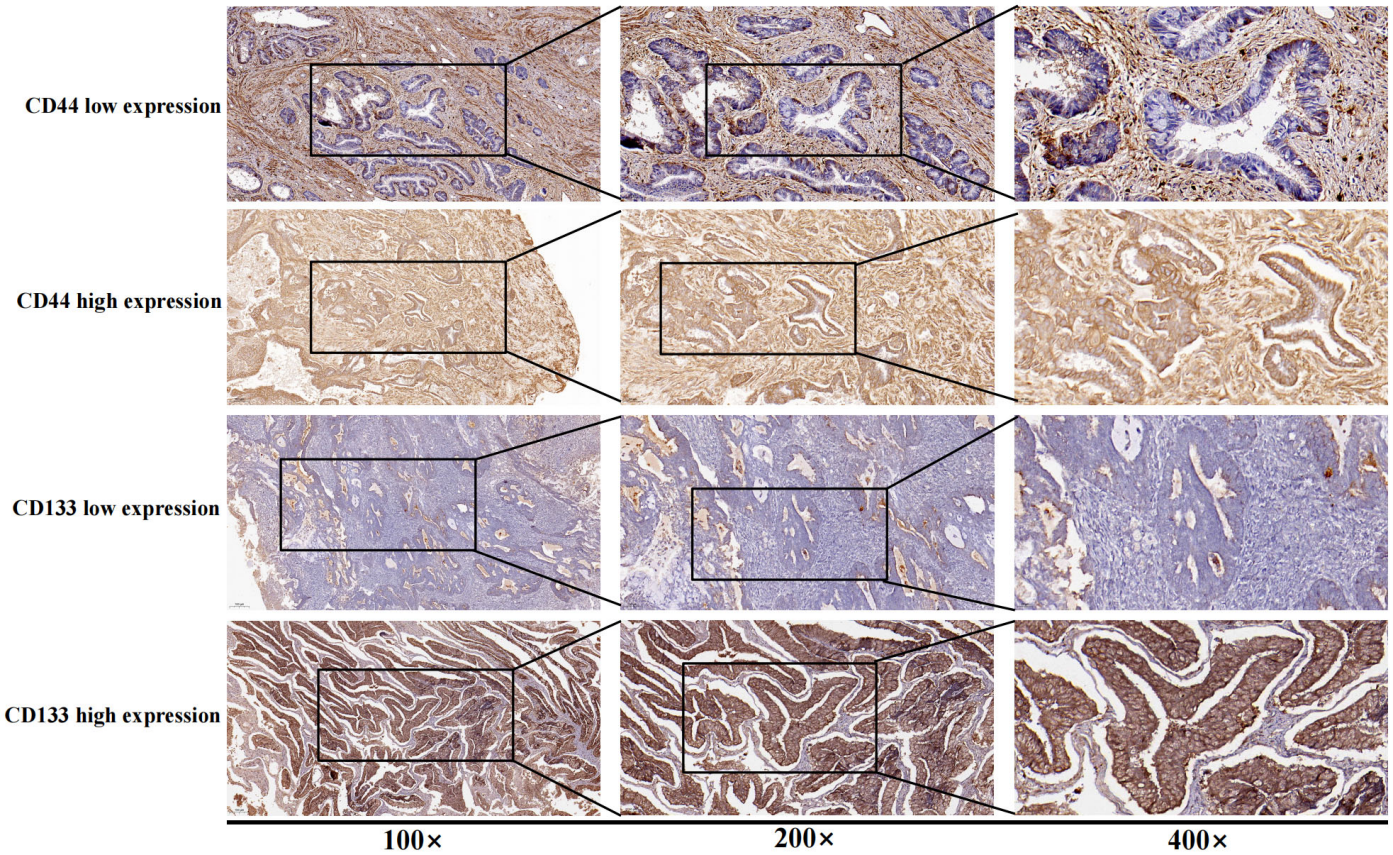
Immunohistochemical staining for low and high expression of CD44 and CD133.

According to the criteria of P53 immunohistochemical interpretation and previous published articles, the immunohistochemical expression results of P53 were divided into abnormal expression (overexpression and completely negative expression) and normal expression (wild-type expression) (18, 19). For the immunohistochemical interpretation results of CD44 or CD133, the proportion of strongly positive staining cells (strongly positive staining on the membrane or cytoplasm of tumor cells) ≥ 50% was defined as high expression of CD44 or CD133, while other situations (including completely negative expression, the proportion of positive tumor cells <50%, and the proportion of tumors cells with medium or weak positive ≥ 50%) were defined as low expression of CD44 or CD133 (**Figure 1**). The above process was independently evaluated by two professional pathologists, if the results of the evaluation were inconsistent, the disputed results were re-evaluated, and a consensus was reached (14).

### 2.3 Definition of co-expression of CD44 & CD133

For the convenience of subsequent research, in this study, simultaneous low expression of CD44 and CD133 was defined as low expression of CD44 & CD133, simultaneous high expression of CD44 and CD133 was defined as high expression of CD44 & CD133, other co-expression situations of CD44 & CD133 (CD44-high but CD133-low expression, CD44-low but CD133-high expression) were defined as mixed expression of CD44 & CD133.

### 2.4 Definition of high-intermediate-risk and high-risk EC patients

According to the 2020 ESGO/ESTRO/ESP guidelines (20), with molecular classification unknown, the following patients were defined as high-intermediate-risk group: (1) Stage I endometrioid + substantial LVSI regardless of grade and depth of invasion; (2) Stage IB endometrioid high-grade regardless of LVSI status; (3) Stage II. The following patients were defined as high-risk group: (1) Stage III-IVA with no residual disease; (2) Stage I-IVA non-endometrioid (serous, clear cell, undifferentiated carcinoma, carcinosarcoma, mixed) with myometrial invasion, and with no residual disease.

### 2.5 Experimental design and statistical analysis

**Figure 2:**
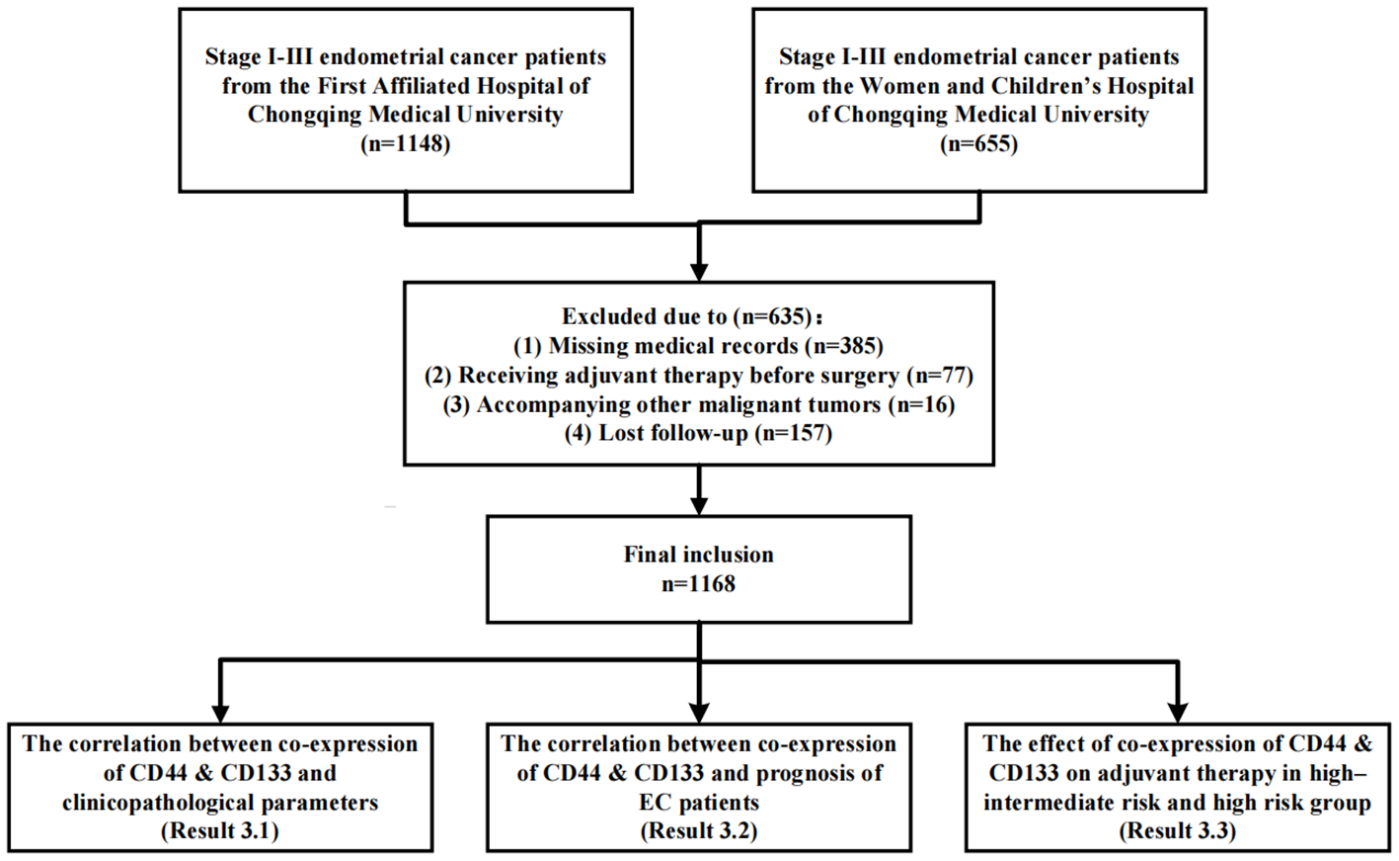
Flow chart of study design and patient inclusion.

The study design was shown in **Figure 2**. Firstly, the Cohen’s kappa coefficient was used to describe the consistency of the expression between CD44 and CD133 (19). The kappa coefficient (k) was mainly used for consistency testing (k<0.01, 0.01-0.20, 0.21-0.40, 0.41-0.60, 0.61-0.80, and 0.81-1.00 indicates no, slight, general, moderate, basic, and almost complete consistency, respectively) (21). The correlation between the co-expression of CD44 & CD133 and clinicopathological parameters was analyzed. Subsequently, both univariate and multivariate Cox regression analyses were conducted to investigate the relationship between the co-expression of CD44 a& CD133 and the prognostic outcomes of EC patients. Factors that exhibited P values less than 0.05 in the univariate analysis were selected for inclusion in the subsequent multivariate analysis. Based on the results of the multivariate analysis, the prognosis models for early-stage (stage I-II) EC patients were constructed, and the model performance was evaluated using receiver operating characteristic (ROC) curve and calibration curve. Ultimately, a stratified analysis was conducted for EC patients categorized as high-intermediate-risk and high-risk, in accordance with the ESGO/ESTRO/ESP guidelines. The Kaplan-Meier survival curves and log-rank tests were applied to assess and compare survival differences between patients who received adjuvant therapy and those who did not, across various groups defined by the co-expression status of CD44 & CD133 (low, mixed, high expression).

Categorical variable was expressed in frequency (%), the chi-square test was used for inter-group comparisons. Continuous variable of normal distribution was expressed in mean (± SD), ANOVA was used for inter-group comparisons. Continuous variable of non-normal distribution was expressed in median (P25, P75), Kruskal Wallis analysis was used for inter-group comparisons. P<0.05 was considered statistically significant. SPSS software (version 26.0, IBM Statistics, Chicago, IL, USA) and R software (version 4.3.1, http://www.r-project.org) were used for data analysis.

## 3 Results

### 3.1 The correlation between the co-expression of CD44 & CD133 and clinicopathological parameters

As shown in **Figure 2**, a total of 1168 patients were ultimately included in this study, with an average age of 53.67 (± 9.30) years. Among them, there were 829 (71.0%), 100 (8.6%), and 239 (20.5%) patients in FIGO stage I, II, and III, respectively. There were 844 (72.3%) patients with pathological type I and 324 (27.7%) patients with pathological type II, respectively (**Table 1**). A cohort of 718 patients received adjuvant therapy, with 374 (52.2%) undergoing radiotherapy exclusively, 39 (5.4%) receiving chemotherapy alone, and 305 (42.4%) being treated with a combination of chemoradiotherapy. The median follow-up time was 44.00 (35.00, 60.00) months. During the follow-up period, 181 (15.5%) patients experienced recurrence, 134 (11.5%) patients died, of which 127 (10.9%) patients died due to recurrence (**Supplementary Table 1**).

**Table 1:** Baseline characteristics of patients and distribution of different co-expression states of CD44 & CD133 in clinicopathological parameters.

Variable	Total patients (n=1168)	Low expression group (n=535)	Mixed expression group (n=344)	High expression group (n=289)	P value
**Age** [yrs, mean (± SD)]	53.67 ( ± 9.30)	53.89 ( ± 9.21)	53.57 ( ± 9.41)	53.39 ( ± 9.35)	0.742
**BMI** [kg/m2, mean (± SD)]	24.58 ( ± 3.69)	24.37 ( ± 3.58)	24.71 ( ± 3.87)	24.80 ( ± 3.65)	0.208
**FIGO stage**					0.017
I	829 (71.0%)	357 (66.7%)	250 (72.7%)	222 (76.8%)	
II	100 (8.6%)	50 (9.3%)	25 (7.3%)	25 (8.7%)	
III	239 (20.5)	128 (23.9%)	69 (20.1%)	42 (14.5%)	
**Pathological type**					0.270
Type I	844 (72.3%)	390 (72.9%)	238 (69.2%)	216 (74.7%)	
Type II	324 (27.7%)	145 (27.1%)	106 (30.8%)	73 (25.3%)	
**Myometrial invasion**					0.017
<1/2	806 (69.0%)	352 (65.8%)	236 (68.6%)	218 (75.4%)	
≥1/2	362 (31.0%)	183 (34.2%)	108 (31.4%)	71 (24.6%)	
**Cervical stromal invasion**					0.100
No	981 (84.0%)	436 (81.5%)	297 (86.3%)	248 (85.8%)	
Yes	187 (16.0%)	99 (18.5%)	47 (13.7%)	41 (14.2%)	
**LVSI**					0.017
Negative	854 (73.1%)	371 (69.3%)	257 (74.7%)	226 (78.2%)	
Positive	314 (26.9%)	164 (30.7%)	87 (25.3%)	63 (21.8%)	
**Lymph node metastasis**					0.067
No	984 (84.2%)	439 (82.1%)	290 (84.3%)	255 (88.2%)	
Yes	184 (15.8%)	96 (17.9%)	54 (15.7%)	34 (11.8%)	
**P53 expression**					0.352
Normal	751 (64.3%)	337 (63.0%)	218 (63.4%)	196 (67.8%)	
Abnormal	417 (35.7%)	198 (37.0%)	126 (36.6%)	93 (32.2%)	

BMI, body mass index; FIGO, International Federation of Gynecology and Obstetrics; LVSI, lymphatic vessel space invasion.

The distribution of CD44 and CD133 expression was shown in **Supplementary Table 2**. In the study population, 289 (24.7%) patients exhibited high expression levels of CD44 & CD133, 535 (45.8%) showed low expression levels, and 344 (29.5%) had mixed expression patterns for these markers. The consistency of the expression between CD44 and CD133 was 70.5% (kappa coefficient was 0.384). As shown in **Table 1**, the proportion of patients with high expression of CD44 & CD133 was higher in patients with early FIGO stage (P=0.017), superficial myometrial invasion (P=0.017), and negative lymphatic vessel space invasion (LVSI) (P=0.017). The distribution of co-expression for CD44 and CD133 did not exhibit significant statistical differences across various clinicopathological parameters, including age, BMI, pathological type, cervical stromal invasion, lymph node metastasis, and P53 expression.

### 3.2 The correlation between co-expression of CD44 & CD133 and prognosis of EC patients

**Figure 3:**
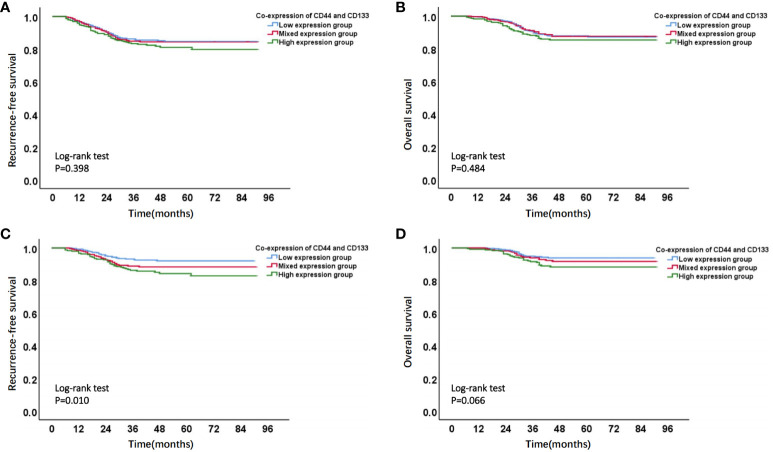
Kaplan-Meier survival curves of different co-expression states of CD44 & CD133 in total (stages I-III, n=1168) and early-stage (stages I-II, n=929) EC patients. **(A)** RFS curve and **(B)** OS curve of different co-expression states of CD44 & CD133 in total (stages I-III, n=1168) EC patients; **(C)** RFS curve and **(D)** OS curve of different co-expression states of CD44 & CD133 in early-stage (stages I-II, n=929) EC patients.

Firstly, Kaplan-Meier analysis found that there was no significant survival difference among different co-expression states (low expression, mixed expression, and high expression) of CD44 & CD133 in total patients (stage I-III, n=1186). However, in early-stage (stage I-II, n=929) patients, compared to patients in low expression group and mixed expression group of CD44 & CD133, patients in high expression group had the worst prognosis. There was a significant difference of RFS rates between groups (P=0.010), and there was a trend of significant difference of OS rates between groups (P=0.066) (**Figure 3** and **Supplementary Table 3**).

In a univariate Cox regression analysis that included the entire patient cohort (stages I-III, n=1186), no significant association was found between the co-expression of CD44 & CD133 and patient prognosis, with P-values of 0.402 for recurrence and 0.481 for death, respectively. However, early-stage (stage I-II, n=929) patients, univariate COX analysis showed a significant correlation between co-expression of CD44 & CD133 and prognosis of patients, further multivariate analysis suggested that co-expression of CD44 & CD133 was still an independent influencing factor for recurrence (P=0.001) and death (P=0.005) of patients. Other factors that were significantly associated with recurrence and death in the multivariate analysis included pathological type, myometrial invasion, cervical stromal invasion, lymphovascular space invasion (LVSI), and P53 expression (**Tables 2**, **3**).

**Table 2:** Univariate and multivariate Cox regression analysis of RFS in early-stage (stages I-II, n=929) EC patients.

Variables	Univariate analysis			Multivariate analysis		
	Hazard ratio	95% CI	P-value	Hazard ratio	95% CI	P-value
**Age** (≥60 vs <60)	1.147	0.723-1.817	0.560			
**BMI**	0.971	0.917-1.028	0.307			
**Pathological type** (Type II vs Type I)	3.625	2.437-5.392	<0.001	2.883	1.913-4.345	<0.001
**Myometrial invasion** (≥1/2 vs <1/2)	3.148	2.118-4.679	<0.001	2.753	1.834-4.132	<0.001
**Cervical stromal invasion** (Yes vs No)	2.115	1.281-3.491	0.003	1.697	1.021-2.822	0.041
**LVSI** (Positive vs Negative)	3.116	2.082-4.662	<0.001	2.387	1.570-3.630	<0.001
**P53 expression** (Abnormal vs Normal)	2.171	1.461-3.226	<0.001	2.019	1.352-3.016	0.001
**Co-expression of CD44 & CD133**						
Low expression	ref		0.012	ref		0.001
Mixed expression	1.546	0.936-2.554	0.089	1.349	0.809-2.250	0.252
High expression	2.073	1.281-3.355	0.003	2.411	1.481-3.926	<0.001

BMI, body mass index; FIGO, International Federation of Gynecology and Obstetrics; LVSI, lymphatic vessel space invasion; ref, reference.

**Table 3:** Univariate and multivariate Cox regression analysis of OS in in early-stage (stages I-II, n=929) EC patients.

Variables	Univariate analysis			Multivariate analysis		
	Hazard ratio	95% CI	P-value	Hazard ratio	95% CI	P-value
**Age** (≥60 vs <60)	1.195	0.690-2.069	0.525			
**BMI**	0.993	0.930-1.061	0.843			
**Pathological type** (Type II vs Type I)	2.930	1.812-4.739	<0.001	2.421	1.479-3.963	<0.001
**Myometrial invasion** (≥1/2 vs <1/2)	3.854	2.391-6.211	<0.001	3.495	2.139-5.708	<0.001
**Cervical stromal invasion** (Yes vs No)	2.483	1.399-4.406	0.002	1.933	1.076-3.472	0.027
**LVSI** (Positive vs Negative)	3.122	1.926-5.061	<0.001	2.343	1.417-3.875	0.001
**P53 expression** (Abnormal vs Normal)	1.911	1.188-3.076	0.008	1.807	1.116-2.923	0.016
**Co-expression of CD44 & CD133**						
Low expression	ref		0.072	ref		0.005
Mixed expression	1.332	0.727-2.440	0.354	1.282	0.692-2.374	0.430
High expression	1.934	1.096-3.413	0.023	2.500	1.402-4.459	0.002

BMI, body mass index; FIGO, International Federation of Gynecology and Obstetrics; LVSI, lymphatic vessel space invasion; ref, reference.

**Figure 4:**
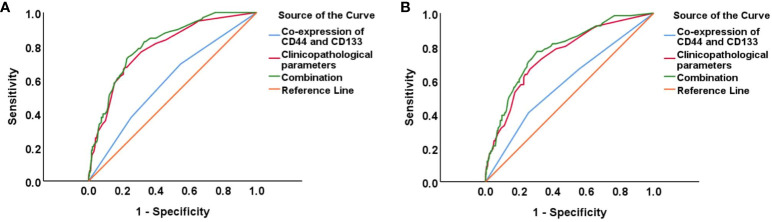
ROC curves for predicting EC **(A)** recurrence and **(B)** death in different groups (CD44 & CD133, clinicopathological parameters, and their combination).

**Figure 5:**
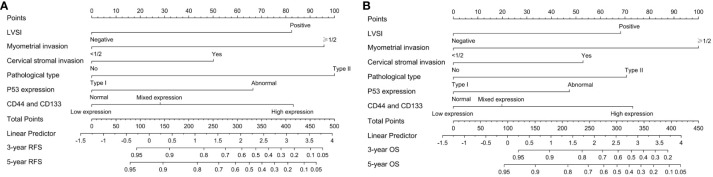
The nomogram models for predicting **(A)** RFS and **(B)** OS of early-stage EC patients based on the co-expression of CD44 & CD133 and clinicopathological parameter.

**Figure 6:**
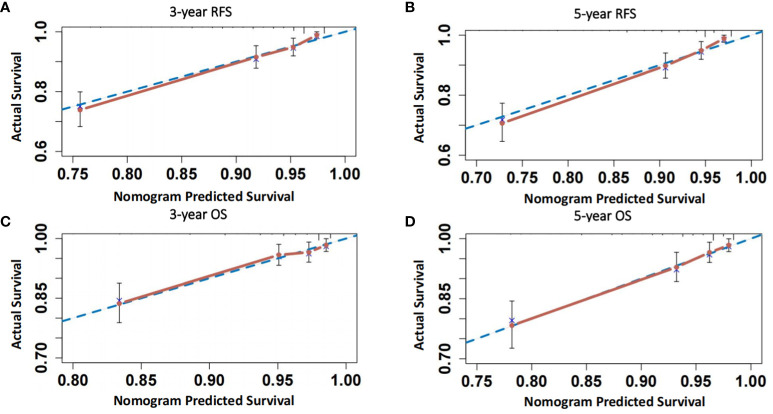
**(A, B)** Calibration curves of the model for predicting 3-year and 5-year RFS of patients; **(C, D)** Calibration curves of the model for predicting 3-year and 5-year OS of patients.

As shown in **Figure 4** and **Supplementary Table 4**, the ROC curve showed that the area under the curve (AUC) of co-expression of CD44 & CD133 for predicting the recurrence and death separately was 0.589 (0.530-0.649) and 0.585 (0.511-0.658), respectively. However, the accuracy of co-expression of CD44 & CD133 combined with clinicopathological parameters in predicting recurrence and death was relatively high, with AUC of 0.810 (0.769-0.851) and 0.777 (0.721-0.834), respectively. To aid in prognostic assessment for patients, we constructed two nomogram models that integrate the co-expression of CD44 & CD133 with clinicopathological parameters, aimed at predicting recurrence and mortality in early-stage patients (refer to **Figure 5**). The calibration curves demonstrated excellent model fit (**Figure 6**).

### 3.3 The effect of co-expression of CD44 & CD133 on adjuvant therapy in high-intermediate-risk and high-risk group

**Figure 7:**
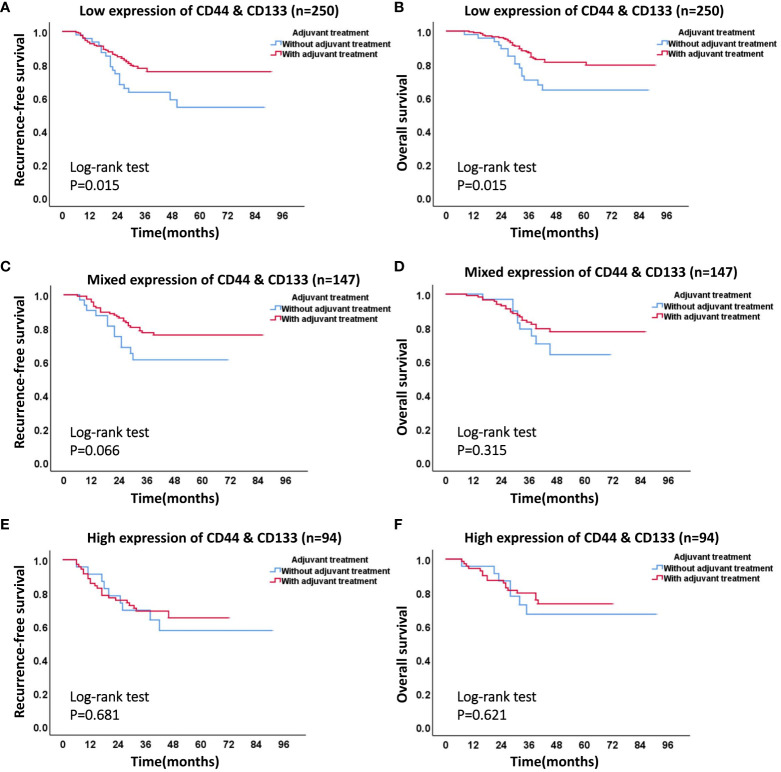
The effect of different co-expression states of CD44 & CD133 on adjuvant therapy in high-intermediate-risk and high-risk group. **(A)** RFS curve and **(B)** OS curve of patients with or without adjuvant therapy in low expression group of CD44 & CD133; **(C)** RFS curve and **(D)** OS curve of patients with or without adjuvant therapy in mixed expression group of CD44 & CD133; **(E)** RFS curve and **(F)** OS curve of patients with or without adjuvant therapy in high expression group of CD44 & CD133.

In accordance with the 2020 ESGO/ESTRO/ESP guidelines, we identified a total of 491 patients in the high-intermediate-risk and high-risk groups. Among these, 389 patients underwent postoperative adjuvant therapy, while 102 patients declined such therapy, citing personal reasons. The stratified analysis was conducted on these patients based on the different co-expression states of CD44 & CD133 (low expression, mixed expression, and high expression). The Kaplan-Meier survival curve showed that, in all three different co-expression groups of CD44 & CD133, patients with adjuvant treatment had higher RFS and OS rates than those without adjuvant treatment. However, only in low expression group, the RFS and OS rates between the groups with and without adjuvant treatment had significant statistical significance (P=0.015 for recurrence and P=0.015 for death, respectively). In mixed expression group, the survival difference between the groups with and without adjuvant treatment decreased, there was only a trend of significant difference in RFS between groups (P=0.066), while no significant statistical difference in OS between groups (P=0.315). In high expression group, the survival difference between the groups with and without adjuvant treatment further narrowed, and there was no significant statistical difference in RFS and OS between groups (P=0.681 for recurrence and P=0.621 for death, respectively) (**Figure 7**, **Table 4**).

**Table 4:** The effect of different co-expression states of CD44 & CD133 on adjuvant therapy in high-intermediate-risk and high-risk group.

Cohort	Group	Number of recurrences	3-year RFS rate (95%CI)	5-year RFS rate (95% CI)	P-value ^a^	Number of deaths	3-year OS rate (95%CI)	5-year OS rate (95%CI)	P-value ^b^
**Low expression group of CD44 & CD133** **(n=250)**	Without adjuvant therapy (n=47)	19	63.3% (49.4%-77.2%)	54.2% (37.5%-70.9%)	0.015	15	70.5% (57.0%-84.0%)	64.5% (49.8%-79.2%)	0.015
	With adjuvant therapy (n=203)	47	77.8% (71.9%-83.7%)	75.7% (69.6%-81.8%)		34	86.9% (82.0%-91.8%)	81.2% (75.3%-87.1%)	
**Mixed expression group of CD44 & CD133** **(n=147)**	Without adjuvant therapy (n=32)	12	61.1% (43.7%-78.5%)	61.1% (43.7%-78.5%)	0.066	9	79.2% (64.3%-94.1%)	63.9% (43.9%-83.9%)	0.315
	With adjuvant therapy (n=115)	26	77.3% (97.3%-99.7%)	75.9% (97.3%-99.7%)		22	83.2% (76.1%-90.3%)	77.6% (69.0%-86.2%)	
**High** **expression group of CD44 & CD133** **(n=94)**	Without adjuvant therapy (n=23)	9	69.6% (50.8%-88.4%)	57.4% (35.6%-79.2%)	0.681	7	67.0% (46.6%-87.4%)	67.0% (46.6%-87.4%)	0.621
	With adjuvant therapy group (n=71)	22	69.1% (58.1%-80.1%)	65.0% (52.1%-77.9%)		17	79.7% (70.1%-89.3)	73.2% (62.0%-84.4%)	

CI, confidence interval; RFS, recurrence-free survival; OS, overall survival; a, log-rank test of RFS; b, log-rank test of OS.

In order to minimize the potential impact of differences in sample size among different groups on statistical results, we took the group with the smallest sample size (high expression group of CD44 & CD133. n=94) as the reference standard, and selected 94 patients with baseline characteristics similar to those in the high expression group from the other two groups through propensity score matching. On the premise of ensuring consistent sample size in each group, survival prognosis analysis was once again used in each group, and the results obtained were consistent with those before propensity score matching (**Supplementary Figure 1**, **Supplementary Table 5**). This indicates that in each group, the impact of adjuvant therapy on patient prognosis is mainly related to the different co-expression states of CD44 & CD133, and is not significantly related to sample size.

## 4 Discussion

As previously discussed, tumor stemness is intimately linked to various cancer phenotypes, including recurrence, metastasis, and drug resistance (22). Laboratory research on tumor stem cells has reached a significant level of maturity; however, the clinical application of tumor stemness markers remains infrequently utilized. CD44 and CD133 serve as classic tumor stem surface antigen markers, which are often used in combination to screen tumor stem cells for various cancers (5). Research has shown that CD133+CD44+ cells were more aggressive in sphere formation, migration, and invasiveness compared with CD133+CD44-, CD133-CD44+, or CD133-CD44- cells (6). Therefore, exploring the prognostic value of co-expression of CD44 and CD133 is more attractive than expression of individual CD44 or CD133. In EC, multiple studies have defined CD44 and CD133 as surface markers of endometrial cancer tumor stem cells. Although significant correlations have been reported between CD44 and CD133, the prognostic value of combined expression of CD44 and CD133 in EC has not been proven (8). In this study, we initially examined the expression consistency between CD44 and CD133 in a dual-center patient cohort. The findings revealed a 70.5% concordance in expression between CD44 and CD133, with a kappa coefficient of 0.384, suggesting moderate agreement between the two markers.

Meanwhile, by analyzing the distribution of co-expression of CD44 & CD133 in clinicopathological features, we found that high expression of CD44 & CD133 was more correlated with early FIGO stage, superficial myometrial invasion, and negative LVSI (**Table 1**), which was basically consistent with the results of a previously reported study (8). However, what has not been highlighted in previous research is that we found early-stage patients with high expression of CD44 & CD133 had a worse prognosis (**Figure 3**; **Supplementary Table 3**). This finding implied that patients with high expression of CD44 & CD133 might not exhibit obvious poor clinicopathological features at the initial stage of tumor development but were still likely to develop distant metastasis or recurrence. It is not difficult to understand, as high co-expression of CD44 & CD133 generally indicates high tumor stemness, tumor cells with high tumor stemness may not necessarily exhibit significant tumor proliferation and infiltration behavior in the initial stage of the disease but may lead to the final progression of the disease by bolstering drug resistance, sustaining self-renewal, and facilitating multi-lineage differentiation (2, 3, 23).

Given that approximately 80% of EC patients are diagnosed in the early-stage (stage I-II), there may be many early EC patients with high expression of CD44 & CD133. Consequently, it is crucial to pay special attention to the prognosis management of these patients. For early-stage EC patients with high expression of CD44 & CD133, vigilance is warranted even in the absence of adverse clinicopathological characteristics. These patients may require meticulous postoperative follow-up and could potentially benefit from timely administration of appropriate adjuvant therapy to mitigate the risks associated with their high tumor stemness. It is imperative to adopt a proactive and comprehensive approach to the treatment and follow-up of early-stage EC patients with high expression of CD44 & CD133 to optimize therapeutic outcomes and minimize the likelihood of disease recurrence or metastatic progression.

In the context of endometrial cancer (EC) patients classified as high-intermediate-risk and high-risk, it is pertinent to discuss the influence of varying co-expression patterns of CD44 & CD133—low, mixed, and high—on the efficacy of adjuvant therapy. Broadly speaking, patients across all three groups who underwent postoperative adjuvant therapy fared better in terms of prognosis compared to those who did not. However, this survival advantage tended to diminish as the co-expression levels of CD44 & CD133 escalated from low to high. Notably, among patients with the highest co-expression levels of these markers, there was no significant difference in survival rates between those who received adjuvant therapy and those who did not. This observation suggests that while adjuvant therapy can positively influence the management of postoperative recurrence in high-risk patients, individuals with high CD44 & CD133 expression may develop resistance to such treatments (24). Consequently, these patients may not reap the full survival benefits offered by adjuvant therapy (25, 26). In light of these findings, for high-intermediate-risk and high-risk EC patients with elevated CD44 & CD133 expression, alternative therapeutic strategies should be contemplated if conventional adjuvant treatments fail to meet expectations. In particular, therapies targeted at tumor stemness, including targeted therapy and immunotherapy, may be considered as viable options to enhance treatment outcomes (26).

At present, molecular classification (POLEmut, MMRd, P53wt, and P53abn) has been gradually applied and promoted in EC due to the disruption of traditional pathological type (27). The 2022 European Society for Medical Oncology (ESMO) guidelines (28) and the 2023 FIGO guidelines (29) have combined existing molecular classification and clinicopathological features to guide prognosis management of EC patients. However, relevant studies have shown that the existing molecular classification systems are not exhaustive and could benefit from the integration of additional molecular markers that hold prognostic significance (27, 30). In our investigation, we assessed the existing molecular subtypes with a focus on P53 expression. Our analysis revealed no substantial link between the co-expression of CD44 & CD133 and P53 expression (P=0.352). However, a marked association was observed between the co-expression of CD44 & CD133 and the clinical outcomes—specifically, recurrence and mortality—among early-stage patients. This suggests that the co-expression of CD44 & CD133 could be regarded as a stand-alone prognostic molecular marker indicative of tumor stemness. To this end, we have integrated the co-expression status of CD44 & CD133 with a range of other clinicopathological factors to develop nomogram models. These models are designed to predict the likelihood of recurrence and death in early-stage endometrial cancer (EC) patients. We are confident that this model will not only aid in the practical application and endorsement of CD44 and CD133 as prognostic indicators for EC but also contribute to the groundwork for the future refinement of molecular classification systems.

The greatest strength of the study was that a larger cohort of patients was obtained through two large medical centers, which fully ensured statistical efficacy. Of course, the study had some shortcomings, such as the fact that it was a retrospective study, and the models developed in the study need to be validated by a prospective external cohort.

## 5 Conclusion

In conclusion, we explored the prognostic value of co-expression of CD44 & CD133 in EC through a dual-center cohort of patients. It is worthwhile for clinicians to be alerted that high expression of CD44 & CD133 is associated with poor prognosis in early-stage patients, and patients with high expression of CD44 & CD133 do not seem to be able to derive significant survival benefit from adjuvant therapy, so these patients may need targeted therapy for tumor stemness.

## Data availability statement

The raw data supporting the conclusions of this article will be made available by the authors, without undue reservation.

## Ethics statement

The studies involving humans were approved by the Institutional Review Board (IRB) of the First Affiliated Hospital of Chongqing Medical University (IRB number: 2021-676) & the Women and Children’s Hospital of Chongqing Medical University (IRB number: 2023-002). The studies were conducted in accordance with the local legislation and institutional requirements. The participants provided their written informed consent to participate in this study.

## Author contributions

PJ: Data curation, Formal analysis, Methodology, Writing – original draft, Writing – review & editing. CT: Data curation, Writing – original draft, Writing – review & editing. YZ: Data curation, Formal analysis, Writing – review & editing. CG: Data curation, Supervision, Writing – review & editing. JW: Data curation, Supervision, Writing – review & editing. YL: Conceptualization, Methodology, Writing – review & editing.
