# Supplementary material for: The prognostic value of co-expression of stemness markers CD44 and CD133 in endometrial cancer
Source: Front Oncol. 2024 Apr 19;14:1338908. doi: 10.3389/fonc.2024.1338908 (PMC11066243; doi:10.3389/fonc.2024.1338908)

**Supplementary Table 1. Adjuvant therapy and follow-up information of patients.**

| **Variable** | **All patients (N = 1168)** |
| --- | --- |
| **Adjuvant treatment** |  |
| Follow-up | 450 (38.5%) |
| Only radiotherapy | 374 (32.0%) |
| Only chemotherapy | 39(3.3%) |
| Chemoradiotherapy | 305(26.1%) |
| **Recurrence** |  |
| No | 987 (84.5%) |
| Yes | 181 (15.5%) |
| **Sites of relapsed (n=181)** |  |
| Vaginal stump | 11 (6.1%) |
| Central pelvic region | 51 (28.2%) |
| Lymph nodes (upper para-aortic) | 21 (11.6%) |
| Peritoneal metastases | 39 (21.5%) |
| Metastasis to other organs | 59 (32.6%) |
| **Death** |  |
| Death of recurrence | 127(10.9%) |
| Death of other reasons | 7 (0.6%) |
| Alive | 1034 (88.5%) |
| **RFS time [months, median (P25, P75)]** | 43.00 (32.00, 59.00) |
| **Follow-up [months, median (P25, P75)]** | 44.00 (35.00, 60.00) |
| **Abbreviations:** RFS, recurrence-free survival; | |

**Supplementary Table 2. The consistency of the expression between CD44 and CD133**

| **Total (n=1168, overall agreement: 70.5%, kappa coefficient: 0.384)** | | | |
| --- | --- | --- | --- |
| **Variable** | **CD133 high expression** | **CD133 low expression** | **Total** |
| **CD44 high expression** | 289 (24.7%) | 183 (15.7%) | 472 (40.4%) |
| **CD44 low expression** | 161 (13.8%) | 535 (45.8%) | 696 (59.6%) |
| **Total** | 450 (38.5%) | 718 (61.5%) | 1168 (100%) |

**Supplementary Table 3. The prognostic distribution of different co-expression states of CD44 & CD133 in total (stages I-III, n=1168) and early-stage (stages I-II, n=929) EC patients**

| **Cohort** | Group | Number of recurrences | 3-year RFS rate (95%CI) | 5-year RFS rate  (95% CI) | P-value ^a^ | Number of deaths | 3-year OS rate  (95%CI) | 5-year OS rate  (95%CI) | P-value ^b^ |
| --- | --- | --- | --- | --- | --- | --- | --- | --- | --- |
| **Total patients**  **(N=1168)** | Low expression group  (n=535) | 77 | 86.2%  (83.3%-89.1%) | 84.7%  (81.6%-87.8%) | 0.398 | 58 | 90.6%  (88.1%-93.1%) | 87.9%  (85.0%-90.8%q) | 0.484 |
|  | Mixed expression group  (n=344) | 52 | 84.8%  (80.9%-88.7%) | 84.4%  (80.5%-88.3%) |  | 37 | 91.1%  (88.0%-94.2%) | 87.6%  (83.7%-91.5%) |  |
|  | High expression group  (n=289) | 52 | 83.3%  (79.0%-87.6%) | 81.0%  (76.1%-85.9%) |  | 39 | 88.2%  (84.5%-91.9%) | 85.4%  (81.1%-89.7%) |  |
| **Stage I-II patients**  **(N=929)** | Low expression group  (n=407) | 30 | 93.2%  (90.7%-95.7%) | 92.1%  (89.4%-94.8%) | 0.010 | 22 | 95.0%  (92.8%-97.2%) | 93.9%  (91.4%-96.4%) | 0.066 |
|  | Mixed expression group  (n=275) | 31 | 88.9%  (85.2%-92.6%) | 88.4%  (84.5%-92.3%) |  | 20 | 93.9%  (91.0%-96.8%) | 91.7%  (88.2%-95.2%) |  |
|  | High expression group  (n=247) | 37 | 86.3%  (82.0%-90.6%) | 84.3%  (79.4%-89.2%) |  | 26 | 91.6%  (88.1%-95.1%) | 88.3%  (84.0%-92.6%) |  |
| Abbreviations: CI, confidence interval; RFS, recurrence-free survival; OS, overall survival; a, log-rank test of RFS; b, log-rank test of OS. | | | | | | | | | |

**Supplementary Table 4. The AUC of different groups for predicting the prognosis of EC**

| **Group** | **AUC (95%CI)** | |
| --- | --- | --- |
|  | **Recurrence** | **Death** |
| **Co-expression of CD44 and CD133** | 0.589  (0.530-0.649) | 0.585  (0.511-0.658) |
| **Clinicopathological parameters**  (Pathological type+ Myometrial invasion + Cervical stromal invasion+LVSI+P53 expression) | 0.786  (0.739-0.833) | 0.750  (0.689-0.811) |
| **Combination**  (Co-expression of CD44 and CD133+ Clinicopathological parameters) | 0.810  (0.769-0.851) | 0.777  (0.721-0.834) |
| **Abbreviations:** AUC, area under the curve. | | |

**Supplementary Table 5. The effect of different co-expression states of CD44 & CD133 on adjuvant therapy in high-intermediate-risk and high-risk group after propensity score matching**

| **Cohort** | Group | Number of recurrences | 3-year RFS rate (95%CI) | 5-year RFS rate  (95% CI) | P-value ^a^ | Number of deaths | 3-year OS rate  (95%CI) | 5-year OS rate  (95%CI) | P-value ^b^ |
| --- | --- | --- | --- | --- | --- | --- | --- | --- | --- |
| **Low expression group of CD44 & CD133**  **(n=94)** | Without adjuvant therapy  (n=23) | 11 | 52.2%  (31.8%-72.6%) | 52.2%  (31.8%-72.6%) | 0.015 | 8 | 64.5%  (44.7%-84.3%) | 64.5%  (44.7%-84.3%) | 0.046 |
|  | With adjuvant therapy  (n=71) | 16 | 77.2%  (87.0%-67.4%) | 77.2%  (87.0%-67.4%) |  | 11 | 85.5%  (77.1%-93.9%) | 83.8%  (75.0%-92.6%) |  |
| **Mixed expression group of CD44 & CD133**  **(n=94)** | Without adjuvant therapy  (n=23) | 9 | 60.3%  (40.1%-80.5%) | 60.3%  (40.1%-80.5%) | 0.105 | 7 | 77.4%  (60.0%-94.8%) | 64.3%  (42.2%-86.4%) | 0.326 |
|  | With adjuvant therapy  (n=71) | 16 | 76.3%  (86.5%-66.1%) | 76.3%  (86.5%-66.1%) |  | 13 | 85.1%  (76.5%-93.7%) | 77.9%  (66.7%-89.1%) |  |
| **High**  **expression group of CD44 & CD133**  **(n=94)** | Without adjuvant therapy  (n=23) | 9 | 69.6%  (50.8%-88.4%) | 57.4%  (35.6%-79.2%) | 0.681 | 7 | 67.0%  (46.6%-87.4%) | 67.0%  (46.6%-87.4%) | 0.621 |
|  | With adjuvant therapy group  (n=71) | 22 | 69.1%  (58.1%-80.1%) | 65.0%  (52.1%-77.9%) |  | 17 | 79.7%  (70.1%-89.3) | 73.2%  (62.0%-84.4%) |  |
| **Abbreviations:** CI, confidence interval; RFS, recurrence-free survival; OS, overall survival; a, log-rank test of RFS; b, log-rank test of OS. | | | | | | | | | |

**Supplementary Figure 1.**

**Caption:** The effect of different co-expression states of CD44 & CD133 on adjuvant therapy in high-intermediate-risk and high-risk group after propensity score matching (n=94 for each group)

**Description:** (A) RFS curve and (B) OS curve of patients with or without adjuvant therapy in low expression group of CD44 & CD133; (C) RFS curve and (D) OS curve of patients with or without adjuvant therapy in mixed expression group of CD44 & CD133; (E) RFS curve and (F) OS curve of patients with or without adjuvant therapy in high expression group of CD44 & CD133.


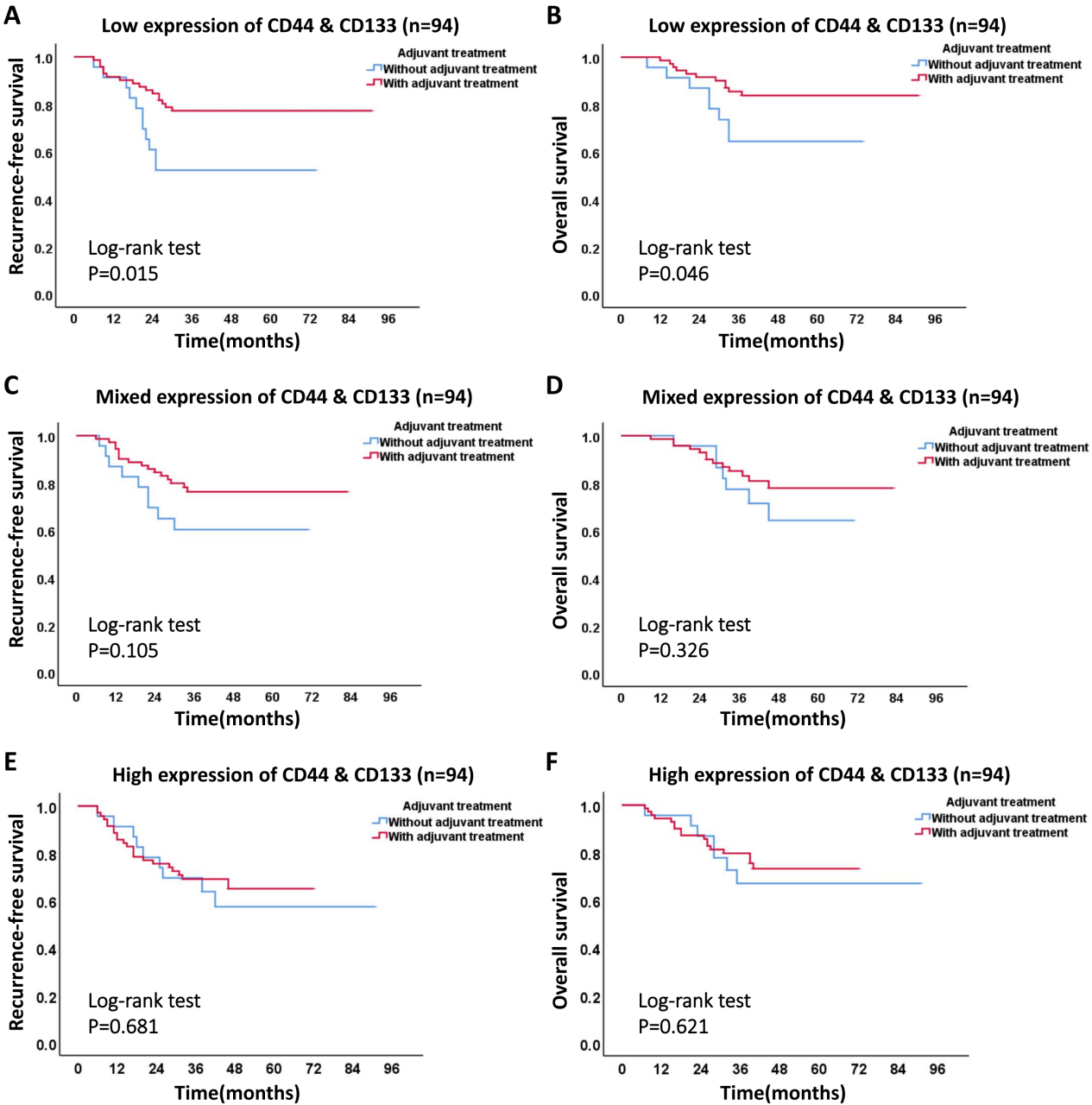

Supplement: Supplementary file 1 [file DataSheet_1.docx]
